# Fracture Strength of Aged Monolithic and Bilayer Zirconia-Based Crowns

**DOI:** 10.1155/2015/418641

**Published:** 2015-10-21

**Authors:** Deborah Pacheco Lameira, Wilkens Aurélio Buarque e Silva, Frederico Andrade e Silva, Grace M. De Souza

**Affiliations:** ^1^Prosthodontics and Periodontology Department, Faculty of Dentistry, University of Campinas (UNICAMP), 13414-903 Piracicaba, SP, Brazil; ^2^Department of Clinical Sciences, Faculty of Dentistry, University of Toronto, 124 Edward Street, Toronto, ON, Canada M5G 1G6

## Abstract

The purpose of this study was to evaluate the effect of design and surface finishing on fracture strength of yttria-tetragonal zirconia polycrystal (Y-TZP) crowns in monolithic (1.5 mm thickness) and bilayer (0.8 mm zirconia coping and 0.7 mm porcelain veneer) configuration after artificial aging. Bovine incisors received crown preparation and Y-TZP crowns were manufactured using CAD/CAM technique, according to the following groups (*n* = 10): Polished monolithic zirconia crowns (PM); Glazed monolithic zirconia crowns (GM); Bi-layer crowns (BL). Crowns were cemented with resin cement, submitted to artificial aging in a chewing simulator (2.5 million cycles/80 N/artificial saliva/37°C), and tested for fracture strength. Two remaining crowns referring to PM and GM groups were submitted to a chemical composition analysis to measure the level of yttrium after aging. One-way ANOVA and Tukey's test (*P* = .05) indicated that monolithic zirconia crowns presented similar fracture strength (PM = 3476.2 N ± 791.7; GM = 3561.5 N ± 991.6), which was higher than bilayer crowns (2060.4 N ± 810.6). There was no difference in the yttrium content among the three surfaces evaluated in the monolithic crowns. Thus, monolithic zirconia crowns present higher fracture strength than bilayer veneered zirconia after artificial aging and surface finishing does not affect their fracture strength.

## 1. Introduction

The increase of esthetics' demand has led to the development of metal-free restorations without metallic components [1]. Dental ceramics present numerous favorable characteristics including biocompatibility and excellent potential to simulate the optical characteristics of natural teeth [2, 3]. However, the evaluation of clinical survival rates of posterior all-ceramic crowns and fixed dental prostheses (FDPs) reveals the vulnerability of those systems to various failure modes [4–6]. Therefore, several attempts have been made to improve the fracture strength of all-ceramic restorations, including the use of Yttria-stabilized tetragonal zirconia polycrystal (Y-TZP) due to its higher flexural strength [7] that allows the manufacturing of fixed partial prostheses (FPPs) in areas of high masticatory loads [8].

However, the strength of all-ceramic crowns relies upon the core as well as the veneer material, whereby a bilayer system with a strong and tough Y-TZP core veneered with translucent but brittle porcelain tends to fail prematurely. Moreover, these bilayer systems have several disadvantages including the multistep manufacturing process, low toughness of the veneer material, and weak bonding between veneer layer and coping [6]. Therefore, zirconia prostheses veneered with porcelain rarely undergo framework fracture, and chipping or cracking of the porcelain veneer is the most commonly reported complication [9–12]. The clinical survival rate of zirconia-based veneer restorations can be as high as 79–100% after 5 years [13–15] and chipping of the veneer layer is mostly reported for bilayers crowns in powder build-up technique [16, 17].

The alternative to circumvent all the bilayer systems' disadvantages is to replace the veneer/core bilayer with a monolithic restorative system [6]. Monolithic lithium disilicate fracture resistance appears promising while submerged in a wet environment [18], and its fatigue load-to-failure showed higher values than veneered Y-TZP crowns [19].

Fabricating zirconia monolithic restorations could improve the mechanical stability and increase the range of indications of those prostheses. However, its wear behavior and chemical stability have not yet been fully clarified. Zirconia presents three different crystal configurations depending on the temperature: monoclinic from room temperature to 1170°C; tetragonal from 1170°C to 2370°C; and cubic at temperatures above 2370°C. When cooling after sintering, this material undergoes volume expansion of 3% to 5%, which is related to the transition from tetragonal to monoclinic phase. Nonetheless, many oxides such as calcium (CaO), magnesium (MgO), yttrium (Y_2_O_3_), or ceria (CeO_2_) may be added to zirconia to stabilize the tetragonal and stronger phase at room temperature [20, 21].

The concentration of the stabilizer plays a decisive role in the performance of this material under fatigue and the addition of 2-3 mol% of Y_2_O_3_ results in partially stabilized tetragonal zirconia, which is the most attractive composition for “transformation toughening” [22]. This mechanism is primarily responsible for the superior mechanical properties of zirconia, since it may undergo phase transformation from tetragonal to monoclinic under localized stress, with a subsequent increase of about 4 to 5% of local volume, inhibiting crack propagation [9, 23]. However, due to its metastable nature, zirconia-based materials are susceptible to unfavorable phase transformation at room temperature, and this phenomenon is known as “low temperature degradation” (LTD) [24, 25]. Aging occurs through an uncontrolled slow transformation of superficial grains from tetragonal-to-monoclinic phase in contact with water. This creates surface roughness and formation of microcracks, creating possibilities for water penetration causing further phase transformation and consequent loss of mechanical strength [24–26].

The aging process may induce yttrium loss and compromise the stability of the tetragonal phase of zirconia-based restorations, leading to uncontrolled tetragonal-to-monoclinic transformation [27]. It has been hypothesized that this mechanism occurs as a result of the reaction between water (H_2_O) and yttrium (Y_2_O_3_) to form yttrium hydroxide (Y(OH)_3_), which steadily drains the stabilizer, allowing for local conversion to the monoclinic phase [21, 28]. Apart from the aging controversy, the application of full-contour zirconia restorations is currently discussed as an alternative to bilayer veneered restorations based on the fact that clinical failures are observed mainly in the veneer layer [29]. In spite of reducing the possibility of early fracture by eliminating the weak phase (veneer layer) from the restorative complex, phase transformation is a reason for concern, since the direct contact with saliva under masticatory loads may aggravate the water penetration and crack propagation.

Hence, the purpose of this study is to compare the fracture strength and failure mode of two Y-TZP monolithic systems, either polished or glazed, and bilayer veneered Y-TZP crowns after prolonged artificial aging. The content of yttrium of the monolithic crowns after artificial aging was also investigated. The null hypothesis was that the crown design, monolithic or bilayer, had no effect on fracture strength of aged zirconia crowns.

## 2. Material and Methods

### 2.1. Specimens' Preparation

**Figure 1:**
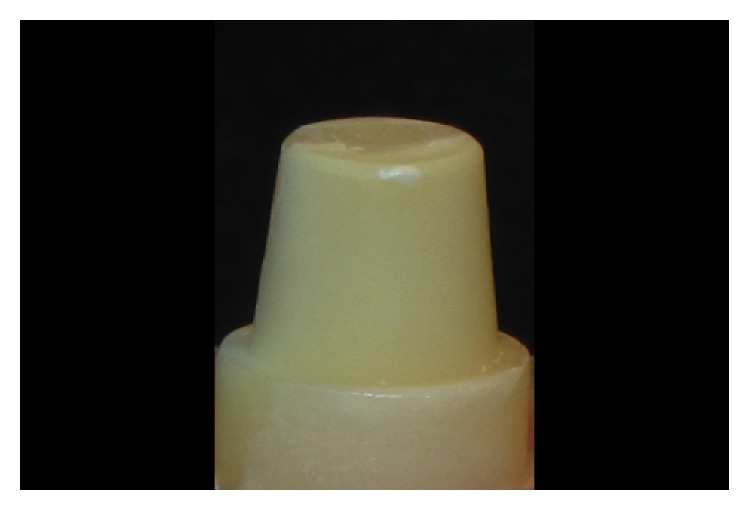
Standardized crown preparation on a bovine incisor.

Thirty-two healthy bovine incisors were used in this study, and a standardized crown preparation was performed in a lathe machine (Magnum-Cut; FEL-2680 GZJ) with the following dimensions: 4.2 mm diameter occlusal base, 6.0 mm diameter cervical base, and 7.0 mm axial height (Figure 1). The taper was established as 8 degrees for all axial walls and the cervical finish line was rounded shoulder. The tooth inner angles were rounded with fine grain diamond burs (KG Sorensen).

Specimens were randomly distributed in three groups (*n* = 10) according to the crown fabrication technique: PM group: monolithic zirconia polished crowns (1.5 mm thickness); GM group: monolithic zirconia glazed crowns (1.5 mm thickness); BL group: zirconia copings with hand-layered porcelain veneering (0.8 mm core and 0.7 mm porcelain thickness). Two additional crowns, referring to PM and GM groups, were submitted to an electron probe microanalysis (EPMA) to quantify the yttrium content after aging.

For fabrication of the nonanatomical crowns, all preparations were scanned by a noncontact optical 3D scanning device (Lava Scan system scanner; 3M ESPE). All zirconia crowns and copings were designed by the same technician with Lava Scan Design System. Then, zirconia blocks (Lava Plus for monolithic crowns, and Lava Frame for by-layer crowns) were milled by using the Lava CNC 500 milling machine (3M ESPE). After the milling procedure, all copings and crowns were sintered in a furnace (Lava Furnace 200) for approximately 11 hours. The fully sintered crowns referring to PM were finished and polished with diamond wheels and bristle brushes (Brasseler; dental instruments). The crowns referring to GM received glaze firing after the sinterization. A silicone impression was taken from one finalized specimen of PM in order to duplicate its 1.5 mm thickness to control the final thickness of the veneered crowns. Copings referring to BL were veneered with the powder build-up technique with Lava Ceram veneer ceramic (3M ESPE). The thickness of the veneered porcelain and the contour of the final crown were verified by measuring the crown at different locations with a digital caliper, and the firing cycle was controlled by an experienced dental technician to ensure standardized crowns.

The crowns were cleaned for 10 min in an ultrasonic bath (Bransonic ultrasonic cleaner 3510 E-DTH; Branson), and 10 specimens of each group were cemented on their respective prepared tooth with a self-adhesive phosphate-based luting resin (RelyX Unicem 2 Automix; 3M ESPE). A static load of 5 kg [11, 30] was applied for 7 minutes following the cementation procedure following the manufacturer's instructions. The crowns for chemical analysis were cemented with temporary cement (RelyX Temp NE; 3M ESPE).

### 2.2. Specimens Aging

After luting, specimens were stored in distilled water at 37°C for 24 hours and submitted to an aging procedure: 2 500 000 cycles, 80 N, at 37°C under artificial saliva bath [31]. Loading was applied with a vertical displacement of 0.2 mm and horizontal (occlusal) displacement of 0.5 mm in a chewing simulator CS-4 (SD Mechatronik). As a substitute for human enamel, hydroxyapatite steatite indenters (3 mm diameter) were used as antagonists and were replaced for each specimen [32].

### 2.3. Fracture Strength Measurement

Aged specimens were loaded in a universal testing machine (Instron; model 8501) under deionized water bath at room temperature, with a 5 mm diameter ball indenter (stainless steel) at a crosshead speed of 0.5 mm/min. The maximum fracture load was measured by applying compressive load to the occlusal surface until the crown failed. Catastrophic fracture failure was considered as either the presence of visible cracks or sudden load drops or even acoustic events of chipping or fracture.

### 2.4. Failure Types' Analysis

The crowns were optically examined after fracture testing, and failure modes were divided into total core fracture, chipping of the veneer, or fracture at core/veneer interface. One representative specimen from each group was mounted on stubs with carbon adhesive tape and colloidal silver paint. Then, specimens were gold-sputtered and observed under scanning electron microscopy (SEM).

### 2.5. Surface Compositional Analysis

The two remaining specimens referring to PM and GM were used for quantification of yttrium content. The yttrium level was measured in 10 points starting from the worn occlusal surface (occlusal dimple) up to the most inner point of the coping of PM and GM and in a surface away from the occlusal load in PM undamaged. Compositional analyses were performed by using electron probe microanalysis (EPMA) on an electron microprobe (camera SX-50/51 DCI 1300 DLL) with 40-degree take-off angle and beam energy of 15 keV.

### 2.6. Statistical Analysis

Fracture strength and yttrium content were separately analyzed by using SPSS 19.0 for Windows (SPSS Inc.). One-way analysis of variance (ANOVA) and Tukey's test were used to compare mean and standard deviation (SD), with 95% confidence levels for both fracture strength and yttrium content.

## 3. Results

### 3.1. Fracture Strength

All crowns withstood the artificial aging in the chewing simulator. One-way ANOVA indicated a significant difference among the groups (*P* = .002, Table 1). The fracture strength of monolithic zirconia crowns polished (PM = 3492.5 ± 748.2 N) and glazed (GM = 3344.7 ± 1159.4 N) was statistically similar (*P* = .930) and significantly (*P* = .002) higher than the results for the bilayer crowns (BL = 2051.8 ± 764.7 N, Table 2).

### 3.2. Failure Types Analysis

**Figure 2:**
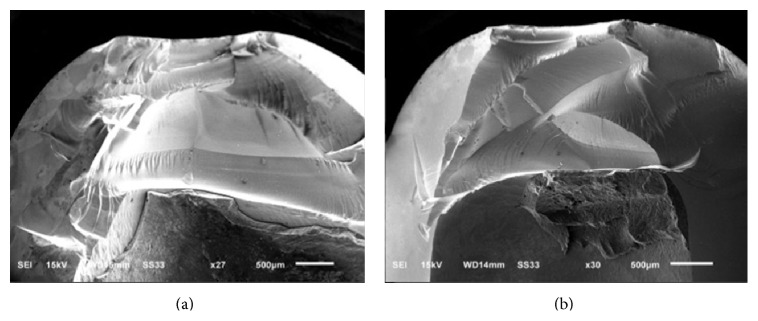
Overview of scanning electron micrographs of polished monolithic crown (PM) ((a) ×27) and glazed monolithic crown (GM) ((b) ×30) fractured specimens.

The failure pattern observed in PM and GM showed total crown fracture (Figure 2). All the specimens from group BL showed fracture at core/veneer interface without infrastructure damage.

**Figure 3:**
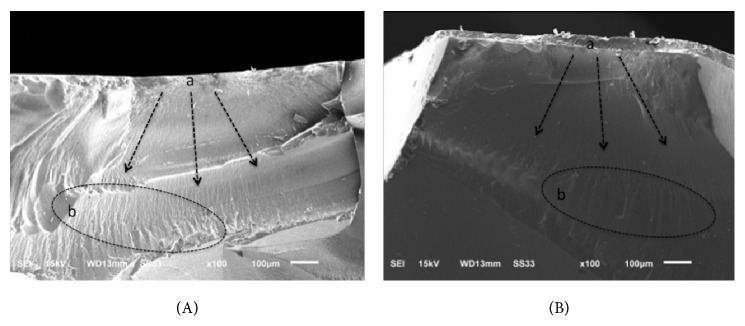
SEM micrographs of polished monolithic (PM) (A) and glazed monolithic (GM) (B) fractured specimens, indicating similar fracture mechanism between them, whereby crack propagation (arrows) starts at occlusal surface (a), and hackles and lines (b) perpendicular to crack origin may be observed.

**Figure 4:**
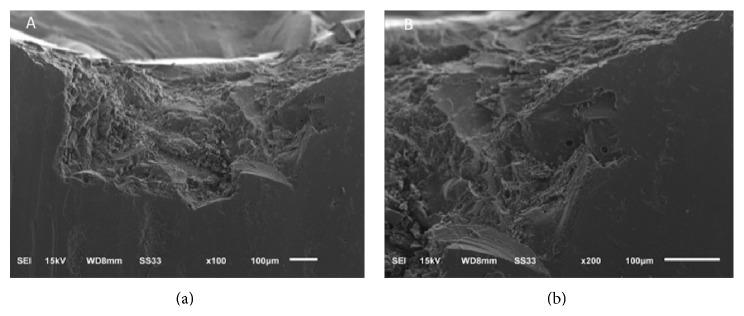
SEM micrograph of porcelain fractured surface showing critical flaw (crack) in bilayer (BL) veneered fractured crown. Note chipping at the occlusal surface (a) and voids inside veneering layer (b).

Fractographic analysis of PM and GM indicates that the direction of the crack propagation occurs from the occlusal surface to the center of the restoration. Based on failure patterns, hackles and lines are perpendicular to the crack origin (Figure 3). In BL, fractographic analysis shows that the critical flaw is located in the middle of the surface damaged inside the veneer layer (Figure 4).

### 3.3. Surface Compositional Analysis

One-way ANOVA of the yttrium content indicated statistically similar (*P* = .935, Table 3) concentration of yttrium among the surfaces. Mean and standard deviation for yttrium content may be observed in Table 4.

## 4. Discussion

The application of artificial aging before the fracture strength test aimed to simulate the effect of the oral environment on zirconia-based crowns by associating cyclic loading, an antagonist tooth, and artificial saliva. This reproduction of the in vivo condition was designed to observe changes representative of the expected clinical in vivo changes, which might result in the undesired phenomenon of low temperature degradation (LTD). 2.5 million mechanical cycles were selected to simulate 5-year aging in the oral environment, considering that an average adult would perform around 500 000 loading cycles/year [33, 34]. However, there is a large variation between number of cycles and the vertical loading applied in aging studies in the literature, with in vitro studies reporting the application of 5 000 to 400 000 cycles [32, 35–39]. Indeed, several studies performed 1 200 000 cycles with 50 N of vertical load [29, 40–42].

All crowns survived the artificial aging in the chewing simulator. This result indicates a stable performance of zirconia-based crowns under a constant load of 80 N during 5 years. Previous studies that evaluated the clinical performance of zirconia-based restorations demonstrated a survival rate of 79–100% after 5 years [13–15], with the most frequent clinical problem being the fracture of the veneering ceramic. Those results may be explained by the uneven masticatory loads presented in vivo, which also varies according to the type of food to be triturated by the posterior teeth. Moreover, other variables are present in the mouth, such as pH and temperature variations, and these effects on the fracture strength and chemical stability of all-ceramic crowns are not well known.

The null hypothesis that the Y-TZP crown design, monolithic or bilayer, has no effect on fracture strength was rejected. Therefore, the present study showed higher fracture strength of monolithic zirconia crowns in comparison to the bilayer configuration.

Previous studies analyzing the fracture strength of all-ceramic monolithic crowns indicate a superior performance for the monolithic design. Monolithic lithium disilicate restorations and hand-layer veneered Y-TZP core evidenced that the highest fatigue load-to-failure values were presented by monolithic crowns and the lowest for veneered Y-TZP crowns [19]. Even though the monolithic system was made of lithium disilicate, better results were obtained when compared to bilayer Y-TZP. According to the authors, the enhanced performance of monolithic crowns may be caused by the elimination of the interface between core and veneer, which is believed to be the weak link in bilayer systems.

Another in vitro study evaluated the load-bearing capacity of four different zirconia based crowns, including zirconia core with veneer layer produced either by powder build-up or CAD/CAM technique, glazed monolithic zirconia, and polished monolithic zirconia. The results showed that zirconia in bilayer configuration had significantly lower load-bearing capacity than the other crowns' design [16]. Nevertheless, it is important to consider that fracture load presented by all groups (PM = 3492.5 N; GM = 3344.7 N; BL = 2051.8 N) was still higher than maximum chewing forces reported in the literature, which is expected to be around 700 N for healthy young adults [43, 44]. Therefore, the results indicated that the fracture load presented by all groups tested in the study may tolerate the clinical applications without restrictions. However, clinical reports of failed bilayer zirconia-based restorations due to chipping or cracking are still commonly reported in the literature [10–12].

In the present study, the groups referring to monolithic crows, polished and glazed, showed a total core fracture pattern. This result was expected, since PM has only one material layer and GM has a thin glaze layer which leads to a bulk structural fracture. On the other hand, all the bilayer crowns showed fracture at core/veneer interface. Failure mode at the veneer layer has been reported for bilayers crowns, most commonly in powder build-up technique rather than in the sintering or pressed veneering technique [16, 17]. This technique, which is highly sensitive and more susceptible to variability due to the individual operator and the many firing cycles required, was used in the present study. The process may result in the addition of impurities and porosities, which maximizes the risk of crack propagation (Figure 3). Therefore, the technique and the low mechanical properties of the veneer material may be the reason for this mode of failure as well as for the lower fracture strength presented by specimens in BL group, since the inner coping was still intact after the mechanical testing. In contrast, there are some researches reporting complete failure (core/veneer) of all Lava CAD/CAM crowns [7] and total coping fracture [17].

There is still no consensus about the system triggering LTD, but three different rationales have been suggested in the literature. The first hypothesis is that water (H_2_O) interacts with yttrium (Y_2_O_3_) generating yttrium hydroxide (Y(OH)_3_), which totally compromises the stabilizer, leading to local yttrium deficiency that results in transformation of tetragonal to monoclinic phase. Another mechanism suggested is that water breaks the bond between Zr and O, resulting in localized stress growth as a result of −OH movement inside the crystal structure. This motion causes lattice fault that acts as nucleating agents for posterior crystalline changes. And the last theory is that O_2_– from water breakdown fills oxygen vacancies [21].

The content of yttrium after aging was evaluated in previous studies. An in vitro study reported yttrium decrease (from 6.76 wt% to 4.83 wt%) after aging Vita In-Ceram YZ in boiling water for 7 days, confirming the first hypothesis for LTD's origin [27]. However, another research with the same experimental method reported no difference in the yttrium content after aging, even showing increase in monoclinic phase concentration (from 2 to 21%) [21]. The contradictory results between the first and the latter references may be related to the distinct chemistry of the zirconia substrates used.

In the current study, there was no difference in the yttrium content among occlusal worn surfaces and undamaged surfaces. Thus, this result can support the hypothesis that the chemical composition of monolithic crowns was not affected by the occlusal loading.

The results of this study demonstrated that monolithic zirconia-based crowns might have reliable fracture strength after 5 years of occlusal loading. Indeed, the fabrication of monolithic zirconia restorations might allow for extended clinical application, reducing a major drawback, which is fracture of veneering ceramic. However, future researches concerning whether temperature or ph variations can influence the fracture strength and chemical stability of monolithic zirconia crowns after artificial aging should be conducted. And in vivo studies should be performed to evaluate the clinical behavior of monolithic zirconia restorations.

## 5. Conclusion

According to the results of this study, Y-TZP monolithic crowns (polished and glazed) present higher fracture strength than bilayered veneered Y-TZP crowns. There was no evidence of yttrium depletion after 2.5 million cycles in artificial aging.

## Figures and Tables

**Table 1:** One-way ANOVA test results for fracture strength effect indicating significant difference amongst the groups.

Source	Sum of squares	df	Mean square	*F*	*P*
Between groups	12563505.80	2	6281752.900	7.571	.002
Within groups	22401284.20	27	829677.193		

Total	34964790.00	29			

**Table 2:** Mean fracture strength and Tukey's test results at 95% significance level.

Experimental group	Fracture strength (*N*)	Std. deviation	Tukey (*P* = .05)
Polished monolithic (PM)	3492.5	748.21	a
Glazed monolithic (GM)	3344.7	1159.45	a
Bilayered veneered (BL)	2051.8	764.76	b^*^

^*^Statistical difference among experimental groups (*P* = .002).

**Table 3:** One-way ANOVA test results for yttrium content, indicating statistically similar values amongst the groups.

Source	Sum of squares	df	Mean square	*F*	*P*
Between groups	.001	2	.000	.067	.935
Within groups	.157	27	.006		

Total	.158	29			

**Table 4:** Mean yttrium content (wt%) in monolithic crowns and Tukey's test results at 95% significance level.

Experimental group	Yttrium content (wt%)	Std. deviation	Tukey (*P* = .05)
PM worn occlusal	2.0785	0.9361	a^*^
GM worn occlusal	2.0822	0.6728	a
PM undamaged	2.0700	0.6443	a

^*^Similar letters indicate statistically similar results among all groups (*P* = .935).
